# Surgery with locking plate or hemiarthroplasty versus nonoperative treatment of 3–4-part proximal humerus fractures in older patients (NITEP): An open-label randomized trial

**DOI:** 10.1371/journal.pmed.1004308

**Published:** 2023-11-28

**Authors:** Antti P. Launonen, Bakir O. Sumrein, Aleksi Reito, Vesa Lepola, Juha Paloneva, Hans E. Berg, Li Felländer-Tsai, Kristo Kask, Timo Rahnel, Kaspar Tootsi, Aare Märtson, Kenneth B. Jonsson, Olof Wolf, Peter Ström, Kaj Døssing, Helle K. Østergaard, Inger Mechlenburg, Ville M. Mattila, Minna K. Laitinen

**Affiliations:** 1 Faculty of Medicine and Health Technology, University of Tampere, Tampere, Finland; 2 Tampere University Hospital, Wellbeing Services County of Pirkanmaa, Tampere, Finland; 3 Department of Surgery, Hospital Nova, Wellbeing Services County of Central Finland, Jyväskylä, Finland; 4 University of Eastern Finland, Kuopio, Finland; 5 Division of Orthopedics and Biotechnology, Department of Clinical Science, Intervention and Technology, Karolinska Institutet, Stockholm, Sweden; 6 Department of Orthopedics, Karolinska University Hospital, Huddinge, Sweden; 7 Department of Orthopaedics, North Estonia Medical Centre, Tallinn, Estonia; 8 Department of Traumatology and Orthopaedics, Tartu University, Tartu, Estonia; 9 Orthopaedics clinic, Tartu University Hospital, Tartu, Estonia; 10 Department of Surgical Sciences, Orthopaedics, Uppsala University, Uppsala, Sweden; 11 Department of Orthopaedic Surgery, Viborg Regional Hospital, Viborg, Denmark; 12 Department of Orthopaedic Surgery, Aarhus University Hospital, Aarhus, Denmark; 13 Department of Clinical Medicine, Aarhus University, Aarhus, Denmark

## Abstract

**Background:**

Proximal humerus fractures (PHFs) are common fractures, especially in older female patients. These fractures are commonly treated surgically, but the consensus on the best treatment is still lacking.

**Methods and findings:**

The primary aim of this multicenter, randomized 3-arm superiority, open-label trial was to assess the results of nonoperative treatment and operative treatment either with locking plate (LP) or hemiarthroplasty (HA) of 3- and 4-part PHF with the primary outcome of Disabilities of the Arm, Shoulder, and Hand (DASH) at 2-year follow-up.

Between February 2011 and December 2019, 160 patients 60 years and older with 3- and 4-part PHFs were randomly assigned in 1:1:1 fashion in block size of 10 to undergo nonoperative treatment (control) or operative intervention with LP or HA. In total, 54 patients were assigned to the nonoperative group, 52 to the LP group, and 54 to the HA group. Five patients assigned to the LP group were reassigned to the HA group perioperatively due to high comminution, and all of these patients had 4-part fractures. In the intention-to-treat analysis, there were 42 patients in the nonoperative group, 44 in the LP group, and 37 in the HA group. The outcome assessors were blinded to the study group.

The mean DASH score at 2-year follow-up was 30.4 (standard error (SE) 3.25), 31.4 (SE 3.11), and 26.6 (SE 3.23) points for the nonoperative, LP, and HA groups, respectively. At 2 years, the between-group differences were 1.07 points (95% CI [−9.5,11.7]; *p* = 0.97) between nonoperative and LP, 3.78 points (95% CI [−7.0,14.6]; *p* = 0.69) between nonoperative and HA, and 4.84 points (95% CI [−5.7,15.4]; *p* = 0.53) between LP and HA. No significant differences in primary or secondary outcomes were seen in stratified age groups (60 to 70 years and 71 years and over).

At 2 years, we found 30 complications (3/52, 5.8% in nonoperative; 22/49, 45% in LP; and 5/49, 10% in HA group, *p* = 0.0004) and 16 severe pain-related adverse events. There was a revision rate of 22% in the LP group.

The limitation of the trial was that the recruitment period was longer than expected due to a high number of exclusions after the assessment of eligibility and a larger exclusion rate than anticipated toward the end of the trial. Therefore, the trial was ended prematurely.

**Conclusions:**

In this study, no benefit was observed between operative treatment with LP or HA and nonoperative treatment in displaced 3- and 4-part PHFs in patients aged 60 years and older. Further, we observed a high rate of complications related to operative treatments.

**Trial Registration:**

ClinicalTrials.gov NCT01246167.

## Abstract 2

In a randomized controlled trial, Antti P Launonen and team compare outcomes of non-operative to operative treatment in patients aged ≥ 60 years with 3-4 part dislocated proximal humerus fractures.

## Author summary

**Why was this study done?:**

- Proximal humerus fractures (PHFs) are among the most common fractures in older adults. The risks for suffering a PHF increase with age, especially after the age of 60 years.
- Nonoperative treatment of non-displaced PHF is uniformly approved, but the treatment of displaced 3- and 4-part PHF remains controversial. Recently, several studies have reported a significant increase in the surgical treatment of the PHF, mainly due to the introduction of locking plates (LPs) suitable for treating osteoporotic bone.
- In this study, we investigated whether surgery with an LP or hemiarthroplasty (HA) is superior to nonoperative treatment with a collar-cuff and early physiotherapy in the treatment of 3- or 4-part PHF involving the surgical neck.

**What did the researchers do and find?:**

- We randomly allocated 160 patients to either nonoperative treatment or operative treatment with HA or LP.
- We recorded the Disabilities of Arm, Shoulder, and Hand (DASH) score, other measures of shoulder function and quality of life, and complications at 2-year follow-up.
- We found no significant or clinically important between-group differences in any of the outcomes.

**What do these findings mean?:**

- This trial provides no evidence that surgery is superior to nonoperative treatment in the treatment of displaced 3- or 4-part PHF involving the surgical neck in older adults.
- These results suggest that the current practice of performing surgery on the majority of displaced 3- or 4-part proximal fractures of the humerus in older adults may not be beneficial.
- The limitation is the trial was ended before intended group sizes.

## Introduction

Proximal humerus fractures (PHFs) are one of the main fractures in the upper extremities sustained by older female patients [1]. According to a Swedish registry study, the incidence of PHF is 175 per 100,000 person-years in adult females [2]. At 48 months after fracture, mortality reaches a peak of up to 24%, which is similar to hip fracture rates, indicating the frailty of patients with PHF [3–6]. Over the past decades, our understanding of the nature of the fracture has changed, and treatment has subsequently evolved. Previously, these fractures were considered to be osteoporotic, and, therefore, research focused solely on the fracture and its morphology [7–11]. However, while trying to find a suitable treatment, the features of frail patients were not always considered.

To date, several attempts have been made to describe the fracture using various current and updated classifications. However, none of these have shown acceptable potential for clinical decision-making, not to mention acceptable reproducibility [12–16]. Moreover, none of the current classification systems can help in deciding on the best treatment for an individual patient. In addition, several studies have reported interobserver variability to be poor at best [17,18]. The introduction of anatomical locking plates (LPs) in the 2000s led to anticipation that restorating the anatomy would improve clinical results. However, subsequent trials showed that LP did not render better clinical results in the older adult population [7,10,19]. Hemiarthroplasty (HA) also became popular, but its use revealed shortcomings, especially tubercle resorption [20], and it was not better than nonoperative treatment [8,9]. In previous randomized controlled trials (RCTs) comparing HA and nonoperative treatment, no superiority of operative treatment could be detected [8,9]. Therefore, a wider understanding of the effects of operative treatments is needed, and the present study aims to fill this knowledge gap with this, to our knowledge, novel 3-arm trial setting.

In a recent Cochrane review, insufficient evidence was found to support the use of any of the operative methods in the treatment of 3- and 4-part PHF [21]. Furthermore, to the best of our knowledge, no previous 3-arm RCT has compared nonoperative treatment with HA and LP in the same setting. The primary aim of this current international multicenter, randomized control trial was to assess the results of the nonoperative treatment of 3- and 4-part PHF compared to operative treatment with either LP or HA with the primary outcome of Disabilities of the Arm, Shoulder, and Hand (DASH) [22].

## Methods

This study was a parallel group, superiority multicenter NITEP (Nordic Innovative Trial to Evaluate Osteoporotic Fractures; www.nitep.eu) group RCT. Patient recruitment was conducted between February 2011 and May 2019 at 7 hospitals in 4 northern European countries: Tampere University Hospital, Finland; Central Finland Central Hospital, Finland; Karolinska University Hospital and Uppsala University Hospitals, Sweden; North Estonia Medical Centre and Tartu University Hospital, Estonia; and Viborg Regional Hospital, Denmark. All sites are primary regional referral centers for orthopedic trauma patients in their respective areas. The initial trial protocol was approved by the Regional Ethics Committee of Tampere University Hospital, Finland. The local ethics committees and the hospital districts provided the research permits before the trial started. The study was conducted in accordance with the Declaration of Helsinki. All patients gave written informed consent prior to participation. Independent steering and monitoring committees observed the trial. The trial was registered at ClinicalTrials.gov (Identifier: NCT01246167).

The original protocol consisted of 2 stratums [23]. Stratum I included 2-part fractures, and Stratum II included 3- and 4-part fractures. The results of Stratum I (2-part fractures) were published in 2019 [24]. The trial design was the work of the members of the protocol committee, and the design and the methods have been published in detail elsewhere [23].

The protocol was signed by the principal investigator (PI) of each site, and the investigator site file (ISF) was held locally using good clinical practice as the gold standard in maintaining the trial. Local researchers and site PIs gathered and managed the data.

The authors who signed the protocol confirmed the accuracy of the reported data, the analysis, and the fidelity of the study to the protocol, with exceptions noted separately. We, the authors, wrote this manuscript and made a joint decision to submit it for publication. The study is reported according to the recommendations of the Consolidated Standards of Reporting Trials (see S1 CONSORT Checklist).

### Patients

Patients aged 60 years and older with displaced 3- and 4-part low-energy PHF sustained within 2 weeks prior to allocation were eligible for inclusion. We used the Neer classification (displacement more than 1 centimeter and/or 45 degrees, with bony contact between all fragments) [25] for classifying the PHFs by assessing every patient’s primary radiographs and computed tomography (CT). Two shoulder trauma surgeons reviewed the radiographs of each patient to ensure the accuracy of the classification.

**Fig 1:**
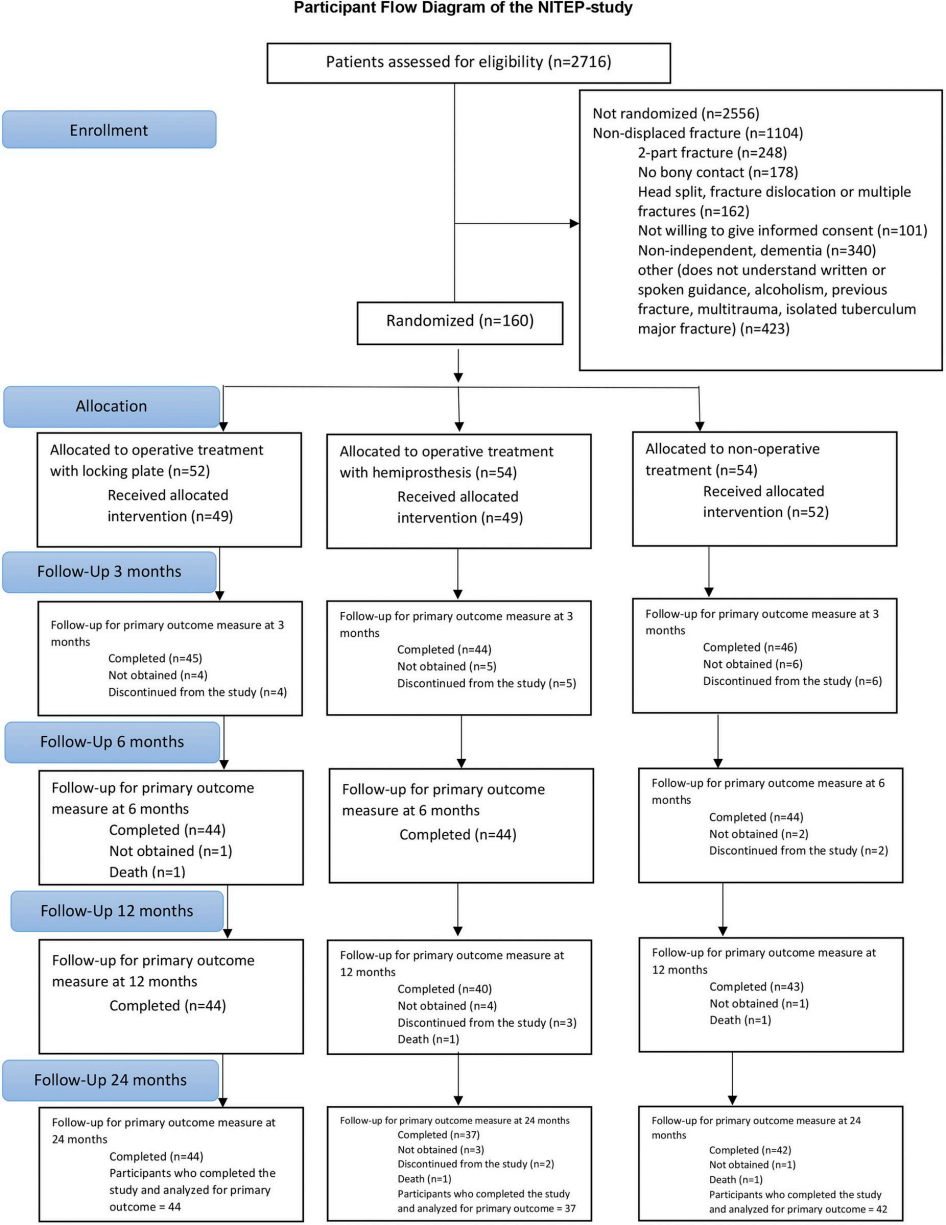
Participant flow diagram of the Nordic Innovative Trial to Evaluate osteoPorotic fractures (NITEP). All patients analyzed according to ITT analysis. Definitions: “Lost to follow-up”–patient could not be reached for the follow-ups; “Not obtained”–Some of the patient data missing, but patient continued in the follow-ups; “Discontinued from the study”–patient wished to discontinue from the trial.

Patients were randomly assigned in a 1:1:1 ratio to undergo nonoperative treatment or operative treatment with LP or HA (Fig 1). After informed consent was obtained, the local researcher contacted the coordinating center’s research coordinator by telephone who then opened the sealed, opaque, and sequentially numbered envelopes in numerical order. The randomization matrix used a block size of 10, according to the center, and stratified by age (60 through 69 years, 70 years and older). We used an open-label, blinded endpoint design: The patients knowing their allocation were encouraged not to reveal it to the outcome assessors who were the physiotherapists or occupational therapists assessing the outcomes (who did not otherwise take part in the study). Further, patients were instructed to wear a shirt to cover any possible scars.

### Surgical and postoperative procedures

The treatment in all groups was standardized. Patients receiving operative treatment had either Philos LP (Synthes, Solothurn, Swizerland) or HA (various brands; see S1 Text) operated by a shoulder orthopedic and trauma surgeon with a minimum of 5 years of experience to prevent learning curve problems. All the groups received the same rehabilitation program (S2 Text). All patients received a written aftercare protocol from physiotherapists with detailed pictures for further rehabilitation in the aftercare period. All patients had 5 face-to-face physiotherapist contacts within the first 3 months after the start of the treatment. Patients in nonoperative and LP treatment had a collar-cuff or a sling was used for 3 weeks to relieve pain. During the first 3 weeks, pendulum exercises were allowed. Active range-of-motion exercises, allowed by pain, began at 3 weeks. Patients treated with a prosthesis wore a sling for 6 weeks. Two weeks postoperatively, they began pendulum movements. Free, active mobilization was allowed at 6 weeks.

### Outcome measures

The primary outcome in this trial was DASH score at 2-year follow-up. The DASH is a patient-reported outcome measure (PROM), which measures self-reported disability and symptoms with 36 questions regarding different daily tasks [22]. The scale ranges from 0 to 100, with the lower end of the scale describing better outcomes. The minimal clinically important difference (MCID) for DASH is determined to be between 10 and 15 points, but our predefined value for clinical importance was set at 10 points [22]. At the time, there was no fracture specific MCID available for DASH. A variety of secondary outcomes were selected in order to help the future meta-analysis and in a result generalizability. As secondary outcomes, we assessed the DASH at 3, 6, and 12 months. Additionally, we used the shoulder specific Constant–Murley Score (CS) [26], the visual analog scale for pain (VAS; 0 to 100 millimeters) [27], the quality-of-life EuroQol Group’s 5-dimension self-reported questionnaire EQ-5D(-3L) [28], another quality-of-life questionnaire 15D [28], and the shoulder-specific Oxford Shoulder Score (OSS) [29]. During the follow-up visits, complications and adverse events (AEs) were recorded. Complications were defined as clinical (infections) or radiological (hardware related, nonunions). AEs were defined as unexpected medical events related to the initial treatment. Serious adverse events (SAEs) were complications requiring further inpatient care.

The patient data acquired were stored in paper portfolios from where they were extracted after the final patient’s 2-year follow-up was completed in December 2021. The portfolios contained the patients’ baseline characteristics, questionnaires, and case report files in which the allocation, collected radiographs, crossovers, complications, AEs, and SAEs were recorded.

In order to avoid the wide interobserver variation reported with the CS, we arranged pretraining sessions for the assessors to standardize the measurements [30]. The questionnaires were administered at baseline and during the follow-up visits at 3, 6, 12, and 24 months after randomization.

### Ethical approval

The initial ethical approval was received from the Regional Ethics Committee of Tampere University Hospital (approval-code R10127). All sites gained local ethical committee approval before the recruitment. Only voluntary patients who gave their informed written consent were included. All data were deidentified.

### Public and patient involvement

When designing the trial, we did not involve patients in trial planning.

### Statistical analysis

The trial was designed to detect an MCID in the DASH score of at least 10 points with a standard seviation (SD) of 18 (effect size d = 0.67). Our pretrial assumption was that the operative treatment arms (LP and HA) would gain an average improvement of 10 points of difference compared to nonoperative treatment (arms 1:1:1), and we used ANOVA (alpha = 0.05, power = 0.8). We assumed a common SD of 18 points and a between-group variance as 25 points. That would have led to an estimated sample size of 66 patients per group, making a total of 198 patients, including an estimated 10% dropout rate. We conducted a preinterim analysis and found no significant differences between the groups. The nonoperative treatment became more common during the study period in the Nordic countries, and in the cases needed, the reverse prosthesis was used. These factors led to a higher-than-expected exclusion rate toward the end of the trial, followed by a premature termination of the trial.

The baseline characteristics were analyzed with descriptive statistics. The primary analysis was carried out with 95% confidence intervals (CIs) after all patients had completed the 2-year follow-up after initial treatment.

The difference between groups was estimated using a linear mixed model allowing for repeated measures. Study group and time of assessment (baseline, 3, 6, 12, 24 months) were included as fixed factors, patients as random factors. The model included interactions between study group and time of assessment. The model was used to estimate the treatment effect as the absolute difference between the groups in DASH score (mean and 95% CIs) and *p*-value at 24 months. Due to the sample size, we used the Satterthwaite method to estimate degrees of freedom [31]. A similar model was used to analyze secondary outcomes where applicable (EQ-5D, 15D, and CS score). For categorical response variables, chi-squared test was used to compare study groups. Because of the potential for type I error due to multiple comparisons, findings for analyses of secondary end points should be interpreted as exploratory. The mixed model allows for missing data. Thus, no data were imputed. We used full analysis population, meaning that all patients having at least 1 posttreatment measurement were included in the analysis.

Continuous outcomes were compared using the Student *t* test. The homogeneity of variance was tested using Levene’s test meeting the assumption of the Student *t* test. Due to the found skewness of the PROMs, we used Mann–Whitney U test to compare the ranks between the groups (against the original plan). All appropriate tests were 2-sided. The analyses were carried out in accordance with a prespecified statistical plan in an intention-to-treat (ITT) manner; the data were analyzed by a randomized group using R software version 4.2.0 (R Foundation for Statistical Computing, Vienna, Austria).

## Results

Between February 2011 and December 2019, a total of 160 patients with 3- and 4-part PHFs were randomly assigned to undergo nonoperative treatment or operative treatment with LP or HA. In total, 54 patients were assigned to the nonoperative group, 52 to the LP group, and 54 to the HA group (Fig 1). Five patients assigned to the LP group were reassigned to the HA group peroperatively due to high comminution, and all of these patients had 4-part fractures. However, all patients were analyzed in their original group in an ITT manner. The characteristics of the included study population are shown in Table 1.

**Table 1:** Characteristics of 160 patients with displaced 3–4-part PHF at baseline.

Treatment groups	Nonoperative *n* = 54, (34%)	LP *n* = 52, (32%)	HA *n* = 54, (34%)
Mean age (SD, range)	70 (6.8, 60–91)	69 (7.6, 62–92)	69 (7.8, 62–90)
Age group, years			
60–69	14 (26%)	13 (25%)	14 (26%)
≥70	40 (74%)	39 (75%)	40 (74%)
Female sex	48 (89%)	43 (83%)	47 (87%)
Fracture: 3-part fracture	36 (67%)	32 (62%)	31 (58%)
Fracture on dominant side	27 (50%)	24 (47%)	24 (45%)
Smoking	5 (9%)	5 (10%)	8 (15%)
Diabetes	8 (15%)	10 (19%)	7 (13%)
Neurological disease	5 (9%)	1 (2%)	0 (0%)
Respiratory disease	8 (15%)	5 (10%)	5 (9%)
Cardiovascular disease	32 (60%)	29 (56%)	29 (54%)
Renal diseases	1 (2%)	1 (2%)	3 (6%)
Hepatobiliary diseases	0 (0%)	0 (0%)	1 (2%)
Psychiatric disorder	2 (4%)	1 (2%)	1 (2%)
Epilepsy	0 (0%)	0 (0%)	1 (2%)
Mean DASH score (SE)	22.8 (3.10)	20.7 (3.23)	20.3 (3.29)
Mean OSS (SE)	39.4 (1.47)	40.4 (1.51)	42.4 (1.51)
Mean EQ-5D score (SE)	0.824 (0.016)	0.838 (0.016)	0.870 (0.016)
Mean 15D score (SE)	0.861 (0.015)	0.867 (0.015)	0.895 (0.015)

DASH, Disabilities of the Arm, Shoulder, and Hand; EQ-5D, EuroQol Group’s 5-dimension self-reported questionnaire; HA, hemiarthroplasty; LP, locking plate; OSS, Oxford Shoulder Score; PHF, proximal humerus fracture; SD, standard deviation; SE, standard error; 15D, 15-dimensional quality-of-life questionnaire.

### Primary outcome

**Fig 2:**
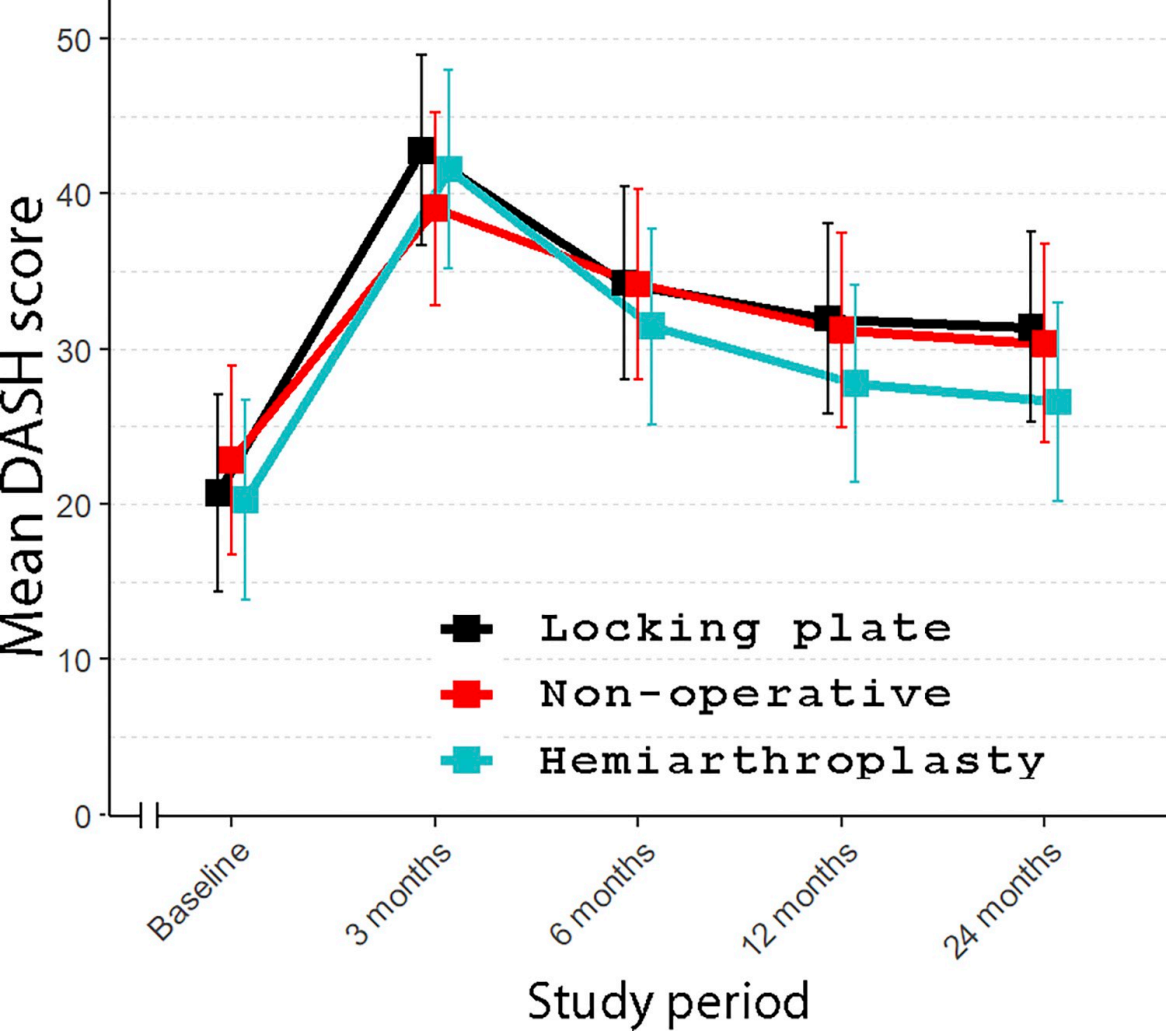
Between-group differences in mean DASH score (0 representing the best, 100 the worst) from baseline to 24-month follow-up. (Vertical lines represent 95% confidence intervals. DASH, Disabilities of the Arm, Shoulder and Hand).

**Fig 3:**
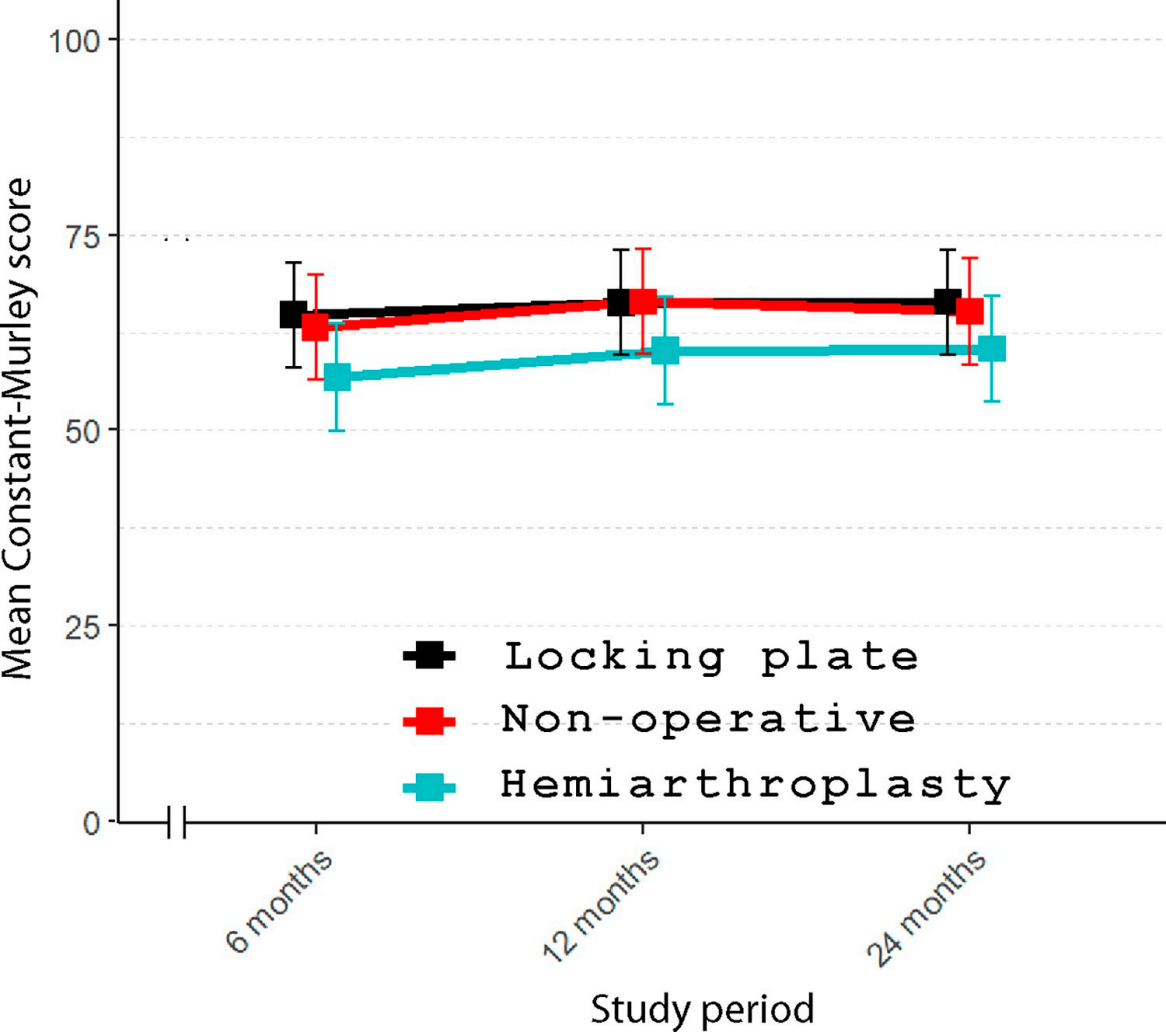
Between-group differences in mean Constant–Murley Score (100 representing the best, 0 the worst) from 6- to 24-month follow-up. (Vertical lines represent 95% confidence intervals).

The mean DASH score at 2-year follow-up was 30.4 (standard error (SE) 3.25), 31.4 (SE 3.11), and 26.6 (SE 3.23) points for the nonoperative, LP, and HA groups, respectively. At 24 months, the between-group differences were 1.07 points (95% CI [−9.5, 11.7]; *p* = 0.97) between nonoperative and LP, 3.78 points (95% CI [−7.0, 14.6]; *p* = 0.69) between nonoperative and HA, and 4.84 points (95% CI [−5.7, 15.4]; *p* = 0.53) between LP and HA. Detailed results are shown in Figs 2 and 3 and Table 2. At 2-year follow-up, there were no significant differences among any of the study groups.

**Table 2:** Outcome measures of NO treated and surgically treated with LP or HA at baseline, 6, 12, and 24 months after displaced 3–4-part PHF.

Evaluation	Nonoperative mean (SE)	LP mean (SE)	HA mean (SE)	Mean difference (95% CI)	*p*
**DASH score**					
**Baseline**	22.8 (3.10)	20.7 (3.23)	20.3 (3.29)		
LP vs. NO				−2.09 (−12.6 to 8.5)	0.89
LP vs. HA				0.45 (−10.4 to 11.3)	0.99
HA vs. NO				2.54 (−8.1 to 13.2)	0.84
**3 months**	39.0 (3.17)	42.8 (3.12)	41.6 (3.23)		
LP vs. NO				3.76 (−6.7 to 14.2)	0.67
LP vs. HA				1.22 (−9.4 to 11.8)	0.96
HA vs. NO				−2.54 (−13.2 to 8.1)	0.84
**6 months**	34.2 (3.11)	34.3 (3.15)	31.5 (3.21)		
LP vs. NO				0.08 (−10.3 to 19.5)	0.99
LP vs. HA				2.80 (−7.8 to 13.4)	0.81
HA vs. NO				2.72 (−7.8 to 13.2)	0.82
**12 months**	31.2 (3.19)	32.0 (3.13)	27.7 (3.23)		
LP vs. NO				0.76 (−9.8 to 11.3)	0.98
LP vs. HA				4.21 (−6.4 to 14.8)	0.62
HA vs. NO				3.45 (−7.2 to 14.1)	0.73
**24 months**	30.4 (3.25)	31.4 (3.11)	26.6 (3.23)		
LP vs. NO				1.07 (−9.5 to 11.7)	0.97
LP vs. HA				4.84 (−5.7 to 15.4)	0.53
HA vs. NO				3.78 (−7.0 to 14.6)	0.69
**CS**					
**6 months**	63.2 (3.39)	64.8 (3.40)	56.8 (3.45)		
LP vs. NO				1.60 (−9.8 to 13.0)	0.94
LP vs. HA				7.97 (−3.5 to 19.4)	0.23
HA vs. NO				6.37 (−5.1 to 17.8)	0.39
**12 months**	66.4 (3.39)	66.3 (3.39)	60.1 (3.47)		
LP vs. NO				−0.11 (−11.5 to 11.2)	0.99
LP vs. HA				6.21 (−5.3 to 17.7)	0.41
HA vs. NO				6.32 (−5.2 to 17.8)	0.40
**24 months**	65.2 (3.43)	66.4 (3.40)	60.4 (3.45)		
LP vs. NO				1.20 (−10.2 to 12.6)	0.9
LP vs. HA				5.99 (−5.5 to 17.5)	0.43
HA vs. NO				4.78 (−6.7 to 16.3)	0.59
**OSS**					
**Baseline**	39.4 (1.47)	40.4 (1.51)	42.4 (1.51)		
LP vs. NO				0.96 (−4.02 to 5.93)	0.89
LP vs. HA				−2.02 (−7.05 to 3.02)	0.61
HA vs. NO				−2.97 (−7.94 to 2.00)	0.34
**3 months**	27.6 (1.56)	28.5 (1.53)	26.5 (1.59)		
LP vs. NO				0.90 (−4.23 to 6.04)	0.91
LP vs. HA				1.97 (−3.22 to 7.16)	0.65
HA vs. NO				1.07 (−4.18 to 6.31)	0.88
**6 months**	32.6 (1.52)	32.9 (1.54)	33.5 (1.58)		
LP vs. NO				0.30 (−4.79 to 5.39)	0.99
LP vs. HA				−0.67 (−5.87 to 4.50)	0.99
HA vs. NO				−0.98 (−6.14 to 4.17)	0.90
**12 months**	34.3 (1.57)	34.3 (1.54)	35.3 (1.61)		
LP vs. NO				0.07 (−5.11 to 5.24)	0.99
LP vs. HA				−0.96 (−6.19 to 4.28)	0.90
HA vs. NO				−1.02 (−6.31 to 4.27)	0.89
**24 months**	35.8 (1.61)	35.0 (1.53)	36.2 (1.59)		
LP vs. NO				−0.76 (−5.99 to 4.46)	0.94
LP vs. HA				−1.23 (−6.42 to 3.95)	0.84
HA vs. NO				−0.47 (−5.80 to 4.86)	0.99
**VAS**					
**Baseline**	54.6 (3.89)	51.3 (3.94)	47.4 (3.86)		
LP vs. NO				−3.30 (−16.31 to 9.72)	0.82
LP vs. HA				3.89 (−9.07 to 16.85)	0.76
HA vs. NO				7.18 (−5.70 to 20.07)	0.39
**3 months**	33.0 (4.10)	41.9 (3.98)	34.0 (4.06)		
LP vs. NO				8.88 (−4.56 to 22.32)	0.27
LP vs. HA				7.88 (−5.48 to 21.25)	0.35
HA vs. NO				−1.00 (−14.57 to 12.58)	0.98
**6 months**	26.1 (4.03)	31.0 (3.98)	25.8 (3.99)		
LP vs. NO				4.87 (−8.44 to 18.18)	0.67
LP vs. HA				5.23 (−8.02 to 18.48)	0.62
HA vs. NO				0.35 (−12.98 to 13.68)	0.99
**12 months**	17.9 (4.07)	25.0 (4.08)	21.1 (4.06)		
LP vs. NO				7.08 (−6.48 to 20.63)	0.44
LP vs. HA				3.95 (−9.58 to 17.49)	0.77
HA vs. NO				−3.12 (−16.65 to 10.40)	0.85
**24 months**	20.0 (4.36)	25.1 (4.11)	21.2 (4.18)		
LP vs. NO				5.12 (−8.97 to 19.22)	0.67
LP vs. HA				3.94 (−9.84 to 17.73)	0.78
HA vs. NO				−1.18 (−15.38 to 13.01)	0.98
**EQ-5D**					
**Baseline**	0.824 (0.0167)	0.838 (0.0169)	0.870 (0.0166)		
LP vs. NO				0.014 (−0.042 to 0.070)	0.83
LP vs. HA				−0.032 (−0.087 to 0.024)	0.38
HA vs. NO				−0.045 (−0.101 to 0.010)	0.13
**3 months**	0.781 (0.0174)	0.786 (0.0170)	0.768 (0.018)		
LP vs. NO				0.005 (−0.052 to 0.062)	0.98
LP vs. HA				0.018 (−0.039 to 0.076)	0.74
HA vs. NO				0.013 (−0.045 to 0.071)	0.85
**6 months**	0.809 (0.0171)	0.802 (0.0174)	0.799 (0.0174)		
LP vs. NO				−0.007 (−0.064 to 0.051)	0.96
LP vs. HA				0.003 (−0.055 to 0.060)	0.99
HA vs. NO				0.009 (−0.048 to 0.067)	0.92
**12 months**	0.815 (0.0177)	0.821 (0.0172)	0.834 (0.0175)		
LP vs. NO				0.006 (−0.052 to 0.064)	0.97
LP vs. HA				−0.013 (−0.071 to 0.044)	0.85
HA vs. NO				−0.019 (−0.078 to 0.039)	0.72
**24 months**	0.823 (0.0183)	0.813 (0.0174)	0.822 (0.0178)		
LP vs. NO				−0.009 (−0.068 to 0.050)	0.93
LP vs. HA				−0.009 (−0.067 to 0.049)	0.93
HA vs. NO				0.0001 (−0.060 to 0.060)	1.00
**15D**					
**Baseline**	0.861 (0.0153)	0.867 (0.0157)	0.895 (0.0150)		
LP vs. NO				0.006 (−0.046 to 0.0574)	0.96
LP vs. HA				−0.028 (−0.079 to 0.0232)	0.40
HA vs. NO				−0.034 (−0.085 to 0.0169)	0.26
**3 months**	0.847 (0.0174)	0.857 (0.0168)	0.867 (0.0166)		
LP vs. NO				0.009 (−0.047 to 0.0664)	0.92
LP vs. HA				−0.011 (−0.066 to 0.0446)	0.89
HA vs. NO				−0.020 (−0.077 to 0.0362)	0.67
**6 months**	0.853 (0.0161)	0.861 (0.0161)	0.879 (0.0163)		
LP vs. NO				0.007 (−0.046 to 0.0611)	0.94
LP vs. HA				−0.0179 (−0.072 to 0.0359)	0.71
HA vs. NO				−0.025 (−0.079 to 0.0286)	0.51
**12 months**	0.855 (0.0172)	0.862 (0.0166)	0.893 (0.0162)		
LP vs. NO				0.007 (−0.0490 to 0.0637)	0.95
LP vs. HA				−0.0309 (−0.086 to 0.0237)	0.38
HA vs. NO				−0.038 (−0.094 to 0.0174)	0.24
**24 months**	0.843 (0.0170)	0.857 (0.0164)	0.906 (0.0169)		
LP vs. NO				0.0146 (−0.041 to 0.0703)	0.81
LP vs. HA				−0.048 (−0.104 to 0.0073)	0.10
HA vs. NO				−0.063 (−0.119 to -0.0062)	0.03

CI, confidence interval; CS, Constant–Murley Score; DASH, Disabilities of the Arm, Shoulder, and Hand; EQ-5D, EuroQol Group’s 5-dimension self-reported questionnaire; HA, hemiarthroplasty; LP, locking plate; NO, nonoperatively; OSS, Oxford Shoulder Score; PHF, proximal humerus fracture; SE, standard error; VAS, visual analog scale; 15D, 15-dimensional quality-of-life questionnaire.

*P* values are based on linear mixed model.

### Secondary outcomes

There was no difference between the study groups in any of the secondary outcomes: CS, OSS, VAS, EQ-5D, or 15D (Table 2). No significant differences in primary or secondary outcomes were seen in stratified age-groups (60 to 70 years and 71 years and over).

### Adverse events

At 2-year follow-up, we found 30 complications (3/52, 5.8% in nonoperative group; 22/49, 45% in LP group; and 5/49, 10% in HA group, *p* = 0.0004) and 16 severe pain-related AEs. In the nonoperative group, there were 2 nonunions and 1 avascular necrosis (AVN). In addition, 3 patients (5.8%) reported severe pain. Two patients (3.8%) required further surgery due to posttraumatic osteoarthritis and severe pain. In the LP group, there were 2 nonunions, 2 deep infections, 6 AVN, and 12 implant-related complications (intra-articular screw penetration, loss of reduction, and screw cutout). In addition, 7 patients (14%) reported severe pain. Complications and pain-related AEs led to 11 reoperations (22%). In the HA group, there were 4 implant-related complications and 1 deep infection. In addition, 6 patients (12%) reported severe pain-related AEs. Complications and pain-related AEs led to 5 reoperations (10%) (Table 3). In addition, tubercle resorption was detected in 32% of the patients in the HA group.

**Table 3:** AEs at 2-year follow-up by type and group of treatment.

Patient characteristics	Nonoperative *n* = 52	LP *n* = 49	HA *n* = 49
Nonunions	2 (3.8%)	2 (4.1%)	
AVN	1 (1.9%)	6 (12%)	
Deep infections		2 (4.1%)	1 (2.0%)
Implant-related complication		12 (24%)	4 (8.2%)
Severe pain	3 (5.8%)	7 (14%)	6 (12%)
Further surgery	2 (3.8%)	11 (22%)	5 (10%)

AE, adverse event; AVN, avascular necrosis; HA, hemiarthroplasty; LP, locking plate.

SAEs, other than severe pain, occurred in 4 patients. In the LP group, 1 patient had myocardial infarction. In the HA group, 1 patient had a stroke, 1 patient had myocardial infarction, and 1 patient had pneumonia.

## Discussion

This multicenter, assessor-blinded, randomized trial involving adult patients aged 60 years and older with 3- and 4-part PHF revealed that operative treatment with LP or HA was not superior to nonoperative treatment with regard to the primary outcome, DASH, assessed at 24-month follow-up. Moreover, the nonoperative group had significantly fewer complications and AEs leading to reoperations. Although all groups showed significant improvement in primary outcome until 12 months, there was no further improvement at 24 months, which is in contrast to the findings of previous smaller trials [7–9,19].

In this study, we found no between-group differences based on the confidence intervals in any of the used outcome measures, including our primary outcome DASH and secondary outcomes CS, OSS, EQ-5D, 15D, VAS, or mortality.

After completing our previous and present PHF studies, combined with the recent Cochrane review, we can acknowledge that the operative treatment of PHF does not provide added benefits in outcomes compared to nonoperative treatment [21,24,32]. Instead, operative treatment enhances morbidity, increasing complications, AEs, and SAEs.

We detected a high rate of complications of up to 45% in the LP group. However, our complication rates were mainly higher than those reported in the current literature, where rates of up to 29% have been reported [7–10,19,33]. In contrast, in our study, the reoperation rates in the LP group were lower than those reported in a previous study [7]. The tubercle resorption rate of 32% in the HA group was at the same level as previously reported [20]. As there is a high risk for complications and reoperations with LPs, they should not be used with the older adult population. For this reason, and in general, patients should be informed about the low complication rates seen in the nonoperative group in this trial before deciding on different treatment options, preferably based on a shared decision-making [34].

In all 3 treatment methods, the shoulder function, measured with DASH, CS, or OSS, remained clinically worse at 1 and 2 years after the fracture when compared to the prefracture baseline level. Recovery after multipart fracture seems to differ from that of 2-part fractures. Indeed, the results of our previous study showed that patients with 2-part fractures regained the prefracture level of function at 1 year [24].

Interestingly, the best DASH scores but at the same time the poorest CS scores (not exceeding the MCID) were observed in the HA group. The discrepancy between these 2 contradicting results might be explained by the assumption that patients slowly adapt to the poor postfracture function in their shoulder, as a portion of the raw points in CS is formed from muscle strength testing and range of motion [26]. Previous studies and the findings of the present trial show that a substantial number of patients had resorbed tubercles after implanting of the HA prosthesis, resulting in both reduced shoulder muscle strength and range of motion [20]. With DASH, however, as a fully subjective questionnaire showed, patients adapted to the poorer function and activities of daily living, regardless of reduced shoulder function.

A recent study suggests that fracture is a sign not only of bone fragility but also of a frail patient, resembling patients with hip fractures [6]. We can reasonably assume that the PHF is a result of the diminished functioning of a person’s reserves, which highlights the need to concentrate on rehabilitation and overall coping in the patient’s daily living. Patients must be considered as individuals and not only as patients with a fracture. PHF in older patients shows that we are mostly dealing with frailty patients who represent a high risk for mortality after sustaining the fracture.

Some limitations in this trial warrant discussion. We ceased recruitment before the predefined power calculation. The results of the preplanned interim analysis after two-thirds of the recruitment was completed revealed no significant differences between treatment alternatives. The trial recruitment period was longer than expected due to a high number of exclusions after assessment of eligibility, since the nonoperative treatment grew more standard in the Nordic countries during the trial. A strength of the study is the multinational, multicenter nature of this NITEP group collaboration, which gives the results high external validity.

It must be acknowledged that in the study, we only investigated nonoperative, HA, and LP treatment options for 3- and 4-part PHF. These were, however, the most relevant treatment options at the start of the trial. Reverse shoulder arthroplasty (RSA) has emerged as a treatment option but was not included in the present study. Furthermore, the number of RSA operations was not screened during the trial. Nevertheless, the NITEP group has already initiated a trial to investigate the outcomes of RSA in comparison to nonoperative treatment (ClinicalTrials.gov Identifier: NCT03531463).

The main conclusion of the present trial is that no benefit was observed between operative treatment with LP or HA and nonoperative treatment in displaced 3- and 4-part PHFs in patients aged 60 years and older. When compared to the prefracture level, functional outcomes still remain at a lower level 1 and 2 years after the fracture. In addition, we observed a high rate of complications related to operative treatments. Further studies should focus on nonoperative methods of treatment, for example, the rehabilitation of these frail patients.

## Supporting information

S1 CONSORT Checklist Checklist of information to include when reporting a randomized trial assessing nonpharmacologic treatments (NPTs). (DOCX)

S2 CONSORT Checklist Checklist of information reporting RCTs assessing NPTs in Journal Abstract. (DOCX)

S1 Text Hemiarthroplasty brands and their amounts/fractions used throughout the trial. (DOCX)

S2 Text Rehabilitation programs used throughout the trial. (DOCX)

S1 Data Sample size and analysis plan. (DOCX)

## Abbreviations

AE: adverse event
AVN: avascular necrosis
CI: confidence interval
CS: Constant–Murley Score
CT: computed tomography
DASH: Disabilities of the Arm, Shoulder, and Hand
EQ-5D: EuroQol Group’s 5-dimension self-reported questionnaire
HA: hemiarthroplasty
ISF: investigator site file
ITT: intention-to-treat
LP: locking plate
MCID: minimal clinically important difference
NITEP: Nordic Innovative Trial to Evaluate Osteoporotic Fractures
OSS: Oxford Shoulder Score
PHF: proximal humerus fracture
PI: principal investigator
PROM: patient-reported outcome measure
RCT: randomized controlled trial
RSA: reverse shoulder arthroplasty
SAE: serious adverse event
SD: standard deviation
SE: standard error
VAS: visual analog scale
