# Supplementary material for: Surgery with locking plate or hemiarthroplasty versus nonoperative treatment of 3–4-part proximal humerus fractures in older patients (NITEP): An open-label randomized trial
Source: PLoS Med. 2023 Nov 28;20(11):e1004308. doi: 10.1371/journal.pmed.1004308 (PMC10683994; doi:10.1371/journal.pmed.1004308)
Supplement: S1 CONSORT Checklist — (DOCX) [file pmed.1004308.s001.docx]

**2017 CONSORT checklist of information to include when reporting a randomized trial assessing nonpharmacologic treatments (NPTs)***

| **Section** | **Item** | **Standard CONSORT description** | **Extension for Nonpharmacologic Trials** | **Section/Paragraph number** |
| --- | --- | --- | --- | --- |
| **Title and abstract** |  |  |  |  |
|  | 1A | Identification as a randomized trial in the title |  | Title |
|  | 1B | Structured summary of trial design, methods, results, and conclusions (for specific guidance see CONSORT for abstracts) | Refer to CONSORT extension for abstracts for NPT trials | According to CONSORT NPT abstract extension (S1 and S2 CONSORT Checklist) |
| **Introduction**  Background and objectives | | | | |
|  | 2A | Scientific background and explanation of rationale |  | Introduction, section 3 |
|  | 2B | Specific objectives or hypotheses |  | Introduction, section 2-3 |
| **Methods** | | | | |
| Trial design | 3A | Description of trial design (such as parallel, factorial) including allocation ratio | When applicable, how care providers were allocated to each trial group | Methods, section 1, 4 |
|  | 3B | Important changes to methods after trial commencement (such as eligibility criteria), with reasons |  | N/A |
| Participants | 4A | Eligibility criteria for participants | When applicable, eligibility criteria for centers and those performing the interventions | Methods, section 3 |
|  | 4B | Settings and locations where the data were collected |  | Methods, section 1 |
| Interventions† | 5 | The interventions for each group with sufficient details to allow replication, including how and when they were actually administered | Precise details of both the experimental treatment and  comparator | Methods, section 5 and Supplement 2 |
|  | 5A |  | Description of the different components of the interventions and, when applicable, descriptions of the procedure for tailoring the interventions to individual participants | Methods, section 5 and Supplement 2 |
|  | 5B |  | Details of whether and how the interventions were standardized. | Methods, section 5 and Supplement 2 |
|  | 5C |  | Details of whether and how adherence of care providers to the protocol was assessed or enhanced | Methods, Section 7 |
|  | 5D |  | Details of whether and how adherence of participants to interventions was assessed or enhanced | N/A |
| Outcomes | 6A | Completely defined pre-specified primary and secondary outcome measures, including how and when they were assessed |  | Methods, Section 6 |
|  | 6B | Any changes to trial outcomes after the trial commenced, with reasons |  | N/A |
| Sample Size | 7A | How sample size was determined | When applicable, details of whether and how the clustering by care providers or centers was addressed | Methods, Section 10 |
|  | 7B | When applicable, explanation of any interim analyses and stopping rules |  | Methods, Section 10 |
| Randomization Sequence generation | 8A | Method used to generate the random allocation sequence | When applicable, how care providers were allocated to each trial group | Methods, Section 4 |
|  | 8B | Type of randomization; details of any restriction (such as blocking and block size) |  | Methods, Section 4 |
| Allocation concealment mechanism | 9 | Mechanism used to implement the random allocation sequence (such as sequentially numbered containers), describing any steps taken to conceal the sequence until interventions were assigned |  | Methods, Section 4 |
| Implementation | 10 | Who generated the allocation sequence, who enrolled participants, and who assigned participants to their groups |  | Methods, Section 4 |
| Blinding (masking) | 11A | If done, who was blinded after assignment to interventions (for example, participants, care providers, those assessing outcomes) and how | If done, who was blinded after assignment to interventions (e.g., participants, care providers, those administering co-interventions, those assessing outcomes) and how | Methods, Section 4 |
|  | 11B | If relevant, description of the similarity of interventions | If blinded, method of blinding and description of the similarity of interventions† | Methods, Section 4 |
|  | 11C |  | If blinding was not possible, description of any attempts to limit bias | Methods, Section 4 |
| Statistical methods | 12A | Statistical methods used to compare groups for primary and secondary outcomes | When applicable, details of whether and how the clustering by care providers or centers was addressed | Methods, Section 11-12 |
|  | 12B | Methods for additional analyses, such as subgroup analyses and adjusted analyses |  | Methods, Section 11-12 |
| **Results** | | | | |
| Participant flow  (a diagram is strongly recommended) | 13A | For each group, the numbers of participants who were randomly assigned, received intended treatment, and were analyzed for the primary outcome | The number of care providers or centers performing the intervention in each group and the number of patients treated by each care provider or in each center | Figure 1 |
|  | 13B | For each group, losses and exclusions after randomization, together with reasons |  | Figure 1 |
|  | 13C |  | For each group, the delay between randomization and the initiation of the intervention | N/A |
| Implementation of intervention | New item |  | Details of the experimental treatment and comparator as they were implemented | N/A |
| Recruitment | 14A | Dates defining the periods of recruitment and follow-up |  | Results, Section 1 |
|  | 14B | Why the trial ended or was stopped |  | Methods, Section 10 |
| Baseline data | 15 | A table showing baseline demographic and clinical characteristics for each group | When applicable, a description of care providers (case volume, qualification, expertise, etc.) and centers (volume) in each group | Table 1; Methods, Section 5 |
| Numbers analyzed | 16 | For each group, number of participants (denominator) included in each analysis and whether the analysis was by original assigned groups |  | Figure 1; Fig 1 legend; Results, section 1 |
| Outcomes and estimation | 17A | For each primary and secondary outcome, results for each group, and the estimated effect size and its precision (such as 95% confidence interval) |  | Methods, Section 1-2, Table 2, Fig 2-3 |
|  | 17B | For binary outcomes, presentation of both absolute and relative effect sizes is recommended |  | N/A |
| Ancillary analyses | 18 | Results of any other analyses performed, including subgroup analyses and adjusted analyses, distinguishing pre-specified from exploratory |  | Results, Section 3 |
| Harms | 19 | All important harms or unintended effects in each group (for specific guidance see CONSORT for harms) |  | Results, Section 4; Table 3 |
| **Discussion** | | | | |
| Limitations | 20 | Trial limitations, addressing sources of potential bias, imprecision, and, if relevant, multiplicity of analyses | In addition, take into account the choice of the comparator, lack of or partial blinding, and unequal expertise of care providers or centers in each group | Discussion, Section 8 |
| Generalizability | 21 | Generalizability (external validity) of the trial findings | Generalizability (external validity) of the trial findings according to the intervention, comparators, patients, and care providers and centers involved in the trial | Discussion, Section 9 |
| Interpretation | 22 | Interpretation consistent with results, balancing benefits and harms, and considering other relevant evidence |  | Discussion, section 10 |
| **Other information** | | | | |
| Registration | 23 | Registration number and name of trial registry |  | Abstract; Methods, Section 1 |
| Protocol | 24 | Where the full trial protocol can be accessed, if available |  | Reference 23 |
| Funding | 25 | Sources of funding and other support (such as supply of drugs), role of funders |  | Submission form |

CONSORT = Consolidated Standards of Reporting Trials; NPT = nonpharmacologic treatment.

*Additions or modifications to the 2010 CONSORT checklist.

†These items are consistent with the Template for Intervention Description and Replication (TIDieR) checklist.

Cite as: Boutron I, Altman DG, Moher D, Schulz KF, Ravaud P; CONSORT NPT Group. CONSORT Statement for Randomized Trials of Nonpharmacologic Treatments: A 2017 Update and a CONSORT Extension for Nonpharmacologic Trial Abstracts. Ann Intern Med. 2017;167(1):40-47. doi:10.7326/M17-0046
