# Supplementary material for: Surgery with locking plate or hemiarthroplasty versus nonoperative treatment of 3–4-part proximal humerus fractures in older patients (NITEP): An open-label randomized trial
Source: PLoS Med. 2023 Nov 28;20(11):e1004308. doi: 10.1371/journal.pmed.1004308 (PMC10683994; doi:10.1371/journal.pmed.1004308)
Supplement: S2 CONSORT Checklist — (DOCX) [file pmed.1004308.s002.docx]

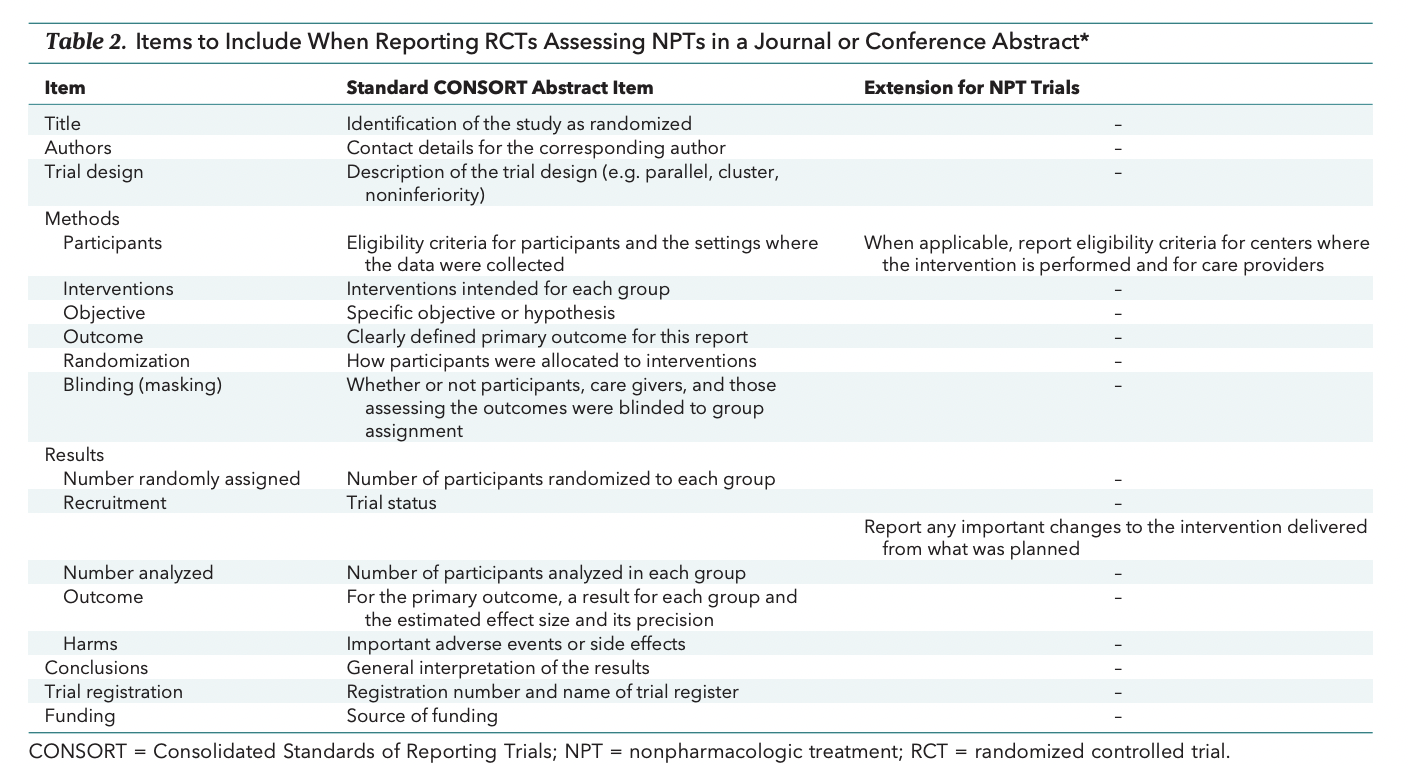


Table referring to CONSORT reporting RCTs assessing NPTs in Journal Abstract

Authors

| **Item** | **CONSORT Abstract** | **Extension for NPT Trials** | **Found in Abstract (Y/N)** |
| --- | --- | --- | --- |
| Title |  |  | title |
| Authors |  |  | Y |
| Trial design |  |  | Y, methods |
| Methods |  |  |  |
| Participants |  |  | Y |
| Objective |  |  | Y |
| Outcome |  |  | Y |
| Randomization |  |  | Y |
| Blinding |  |  | Y |
| Results |  |  |  |
| Number randomly assigned |  |  | Y |
| Recruitment |  |  | Y |
| Number analyzed |  |  | Y |
| Outcome |  |  | Y |
| Harms |  |  | Y |
| Conclusions |  |  | Y |
| Trial registration |  |  | Y |
| Funding |  |  | N |
|  |  |  |  |
