# Supplementary material for: Surgery with locking plate or hemiarthroplasty versus nonoperative treatment of 3–4-part proximal humerus fractures in older patients (NITEP): An open-label randomized trial
Source: PLoS Med. 2023 Nov 28;20(11):e1004308. doi: 10.1371/journal.pmed.1004308 (PMC10683994; doi:10.1371/journal.pmed.1004308)
Supplement: S1 Text — (DOCX) [file pmed.1004308.s003.docx]

**Supplement 1**

Hemiarthroplasty brands and their amounts/fractions used throughout the trial

| **Brand** | **Number of patients** | **percentile** |
| --- | --- | --- |
| Epoca (Depuy Synthes) | 25 | 46% |
| Comprehensive Fx system (Zimmer-Biomet) | 15 | 28% |
| Global Unite (Depuy Synthes) | 8 | 15% |
| Global Fx (Depuy Synthes) | 6 | 11% |
| **total** | 54 | 100% |
