# Supplementary material for: Surgery with locking plate or hemiarthroplasty versus nonoperative treatment of 3–4-part proximal humerus fractures in older patients (NITEP): An open-label randomized trial
Source: PLoS Med. 2023 Nov 28;20(11):e1004308. doi: 10.1371/journal.pmed.1004308 (PMC10683994; doi:10.1371/journal.pmed.1004308)
Supplement: S2 Text — (DOCX) [file pmed.1004308.s004.docx]

**Supplement 2**

Rehabilitation programs were used throughout the trial. We used short immobilisation time and early onset rehabilitation due to previous studies showing that short immobilization may be beneficial to the patient.

**Non-operative treatment-group**

Patients in non-operative treatment were instructed with regard to joint mobilization by a physiotherapist during hospitalization. Patients received a written aftercare protocol from physiotherapists with detailed pictures for further rehabilitation in the aftercare period. A collar-cuff or a sling was used for three weeks to relieve pain. During the first 3 weeks, pendulum exercises were allowed, and free joint mobilization and normal limb activation throughout treatment were strongly supported by the doctors and hospital staff. Active range-of-motion exercises, allowed by pain, began at three weeks. Physiotherapist contacts were arranged to begin at three weeks. All patients had 5 face-to-face physiotherapist contacts within the 3 first months after the start of the treatment. The physiotherapy sessions were arranged during the controls at the hospital and other times at the health-care central.

**Surgery treatment-groups**

Patients operated with a locking plate followed the same protocol as in non-operative treatment.

Patients treated with a prosthesis wore a sling for six weeks. Two weeks postoperatively, they began pendulum movements. Free, active mobilization was allowed at six weeks. Patients were advised to mobilize their free joints from the beginning of the treatment and normal limb activation during aftercare was supported by the doctors and hospital staff. Face-to-face contact with a hospital physiotherapist began after 3 and 6 weeks postoperatively and all patients had 5 face-to-face physiotherapist contacts within 3 months from the beginning of treatment. Patients received a detailed written aftercare protocol with instructional pictures and formal physiotherapy was instructed before leaving the hospital post-operatively.
