# Supplementary material for: Surgery with locking plate or hemiarthroplasty versus nonoperative treatment of 3–4-part proximal humerus fractures in older patients (NITEP): An open-label randomized trial
Source: PLoS Med. 2023 Nov 28;20(11):e1004308. doi: 10.1371/journal.pmed.1004308 (PMC10683994; doi:10.1371/journal.pmed.1004308)
Supplement: S1 Data — (DOCX) [file pmed.1004308.s005.docx]

Sample size and analysis plan

Sample size

The trial is designed to detect a MCID in the DASH score of at least 10 points with a Standard Deviation (SD) of 18 (effect size d=0.67). Our pre-trial assumption was that the operative treatment arms (LP and HA) would gain an average improvement of 10 points of difference compared to non-operative treatment (arms 1:1:1), and we used ANOVA (alpha=0.05, power=0.8). We assumed a common SD of 18 points and a between-group variance as 25 points. That would have led to an estimated sample size of 66 patients per group, making a total of 198 patients, including an estimated 10% drop-out rate. We conducted a pre-interim analysis and found no significant differences between the groups. The non-operative treatment became more common during the study period in the Nordic countries and in the cases needed, the reverse prosthesis was used. These factors led to a higher-than-expected exclusion rate towards the end of the trial, followed by a premature termination of the trial.

Statistical analysis as written in the study protocol

Differences between groups in continuous skewed main outcome variables will be analyzed by the Mann–Whitney U-test and t-test when variables are unskewed. Results are presented with 95% confidence intervals. Two-way-tables with the chi-square test will be used for dichotomous variables. Multivariate analysis will be conducted with regression analysis. In subgroup analysis the effect of age, sex, fracture group, smoking, and other diseases will be evaluated against the ROM, OSS, Constant-Murley, and overall quality of life after fracture.

The final statistical analyses for the publication

We will perform a full data set analysis which includes three months, 6 months, 12 months and 24 months outcomes as independent values. This repeated measures analysis will be performed for DASH, OSS and 15D. For the Constant-Murley score only 6-, 12- and 24-month data are available. Baseline data are not available for the Constant-Murley score because it includes a strength measurement that is not feasible in patients with an acute fracture. Marginal estimates for group-time interactions in each time point are used as the primary numerical results.

The primary statistical method is a linear mixed model. The patient is a random factor and group-time interaction and any covariates will be fixed factors. Marginal effects for group-time interaction will be estimated. The Satterthwaite method is used for degrees of freedom approximation.

Secondary analyses will be done using age as an additional covariate.

lmer function in the lme4 package is used in the linear mixed models. Emmeans package is used to estimate the marginal means and associated p-values and 95% confidence intervals. Chi-square test is used to compare binary and ordinal outcomes at each time-point.

Initial analysis will be done blindly. After blinded interpretation of the numerical results, we will re-run the analysis. All pair-wise comparison will be shown as a priori there is no reference treatment group. All p-values and presented with 95% confidence intervals.

All models used in the analyses will include respective baseline values as covariates*. Age will be added in a secondary analysis.*

Subgroup analyses will be done stratified by age 70 years of age.

We carry on with subgroup analyses other than the effect of age due to small groups.
